# Supplementary material for: On the price dynamics of non-fungible tokens: The ‘Bored Apes’ case
Source: PLoS One. 2023 Nov 16;18(11):e0287881. doi: 10.1371/journal.pone.0287881 (PMC10653520; doi:10.1371/journal.pone.0287881)
Supplement: Attachment — Submitted filename: A Review.docx [file pone.0287881.s003.docx]

Please address the following issues before resubmitting:

1. Your current structure is messy and so please re-structure paper as follows: 1. Introduction, 2. Background, 3. Theoretical literature review, 4. Empirical literature review and hypotheses development, 5. Research design, 6. Empirical results and discussion, and 7. Summary and conclusion. Please you must re-structure your paper according this comment 1, which will involve substantial revisions to your work. To reduce wasting the time of reviewers, yours and mine, if this suggestion is not followed, then, the paper may be rejected without any further review, and so please kindly take this comment seriously.

2. **Introduction:** Please clarify your research questions, objectives, background motivation, theoretical and empirical motivation and the lines of contributions to the literature. You can do this by sharply articulating your research questions/objectives, identify the potential theoretical, background and theoretical motivation or gaps, and explain how your study contributes to the literature. You can do this by highlighting the weaknesses of prior studies as well. Currently, your introduction is very dry. Additionally, you need state clearly the contributions of the paper. For example, “Consequently, the current paper seeks to make the following contributions to the existing literature. First,…, Second,…., Third, …, Fourth,… and so on”. The introduction should be about 5 pages long.

3. **Background** – you need to explain why this is the appropriate context to conduct this study by exploiting regulatory, reform and policy issues and developments within the research context or setting. This should be about two to three pages long.

4. **Theoretical framework** - Please an overarching theoretical framework that will explain the underlying predictions and hypotheses of interest. In doing so, please explicitly outline how they help link the dependent and independent variables together by drawing on both seminal (old) and recently (newly) published studies. This should be about two to three pages long.

5. **Literature review and hypotheses Development** – please enhance your hypotheses by: (i) drawing on the theory; (ii) empirical literature; (iii) research setting/contextual insights; and (iv) then setting up your hypotheses. You will do this for each hypothesis. Currently, you have not developed your hypotheses in this way. You will need to so by drawing on both seminal (old) and recently (newly) published studies.

6. **Research design** – Please identify, classify and explain your variables – dependent, independent and control variables, as well as any others, such as moderating or mediating variables. Please also explain your sample selection clearly (insert a table tabulating the steps - how many was missing, many had data, how many selected and why) and also clarify in a normative way how the variables are operationalised. Similarly, explain your sample in a tabular form, outlining step by step the total population to the selection of the final sample. Label all your equations, figures and tables in a consecutive manner. Make the tables self-contained by clearly identifying dependent, independent and control variables in the tables.

7. **Empirical findings** – please link your findings more strongly to the: (i) theory, (ii) empirics, (iii) context; and (iv) highlight their economic, academic/research and policy implications. Closely link up and cite the papers that you have discussed in the background, theory and empirical literature review & and hypotheses development section to the findings you are presenting here.

8. **Conclusion** – Please outline a summary of findings, contributions, implications, limitations and avenues for future research. Especially, expand the discussions relating to implications, limitations and avenues for future research.

9. **Robustness or additional analyses** – please demonstrate how your findings are to alternative measures (e.g., different ways of measuring the key dependent and independent variables), estimations (e.g., lagged structure, and instrumental variables estimation, amongst others) and general endogeneities. This is completely missing.

I hope you will positively embrace these constructive suggestions as a way of taking this research forward, and I look forward to receiving a revised version of your paper.

Best Regards
