# Supplementary material for: On the price dynamics of non-fungible tokens: The ‘Bored Apes’ case
Source: PLoS One. 2023 Nov 16;18(11):e0287881. doi: 10.1371/journal.pone.0287881 (PMC10653520; doi:10.1371/journal.pone.0287881)
Supplement: Attachment — Submitted filename: HowRef_3.docx [file pone.0287881.s004.docx]

**On the price dynamics of non-fungible tokens: The ‘Bored Apes’ case**

Revised - Revised - Version for *Plos One*

*(Ms:* PONE-D-22-31290 PLOS ONE )

Authors’ response to the comments of the Editor and the Reviewers

First of all, let us thank the Reviewers and the Editor, once again, for their very careful reading of this article and valuable comments. We also thank them for the encouraging evaluations.

We received no comments from Referee 1 and 2 (Referee 2 was satisfied by the changes made in the revised version following his/her previous comments). Referee 3 made no comments regarding the “substance” of the research, while he/she recommended a deep revision of the exposition and presentation, with detailed suggestions regarding the structure of the paper. (However, some suggestions of Referee 3 are in conflict with the opinion of the Referee 1 on the first version of the paper --for instance, concerning the content of the Introduction). In any case, we paid serious attention on the comments of the Referee 3, and the structure of the paper has been changed accordingly.

Specifically, the paper has been re-structured as follows. (1) Introduction, including: (i) a sub-section dealing with the back-ground of the case study; (ii) a sub-section with a review of the relevant literature; (iii) a sub-section explaining the specific contribution of the present study. (2) Theoretical framework and the research design. (3) Data. (4) Empirical findings, articulated in (i) The empirical model for the price dynamics; (ii) Daily seasonality in price level and volatility; (iii) Relations with financial markets; (iv) Relations with the exchange rate. (5) Additional analysis (with the computation of a price index from a hedonic price approach, which is also interpreted as a+ robustness check). (6) Conclusions.

This structure is substantially in line with the suggestion of Referee 3, and also consistent with the observations of Referee 1 in the first stage of the reviewing process.

We are confident that the new structure permits to highlight the contribution of the present analysis in a clear and effective way.

Let us notice that all the data used in the analysis are freely downloadable from the web; in any case, we also up-load the file with the (final) dataset used in the analysis. We can publish this file in open sites (and the journal has been already provided with the file).
